# Non-coding nucleotides and amino acids near the active site regulate peptide deformylase expression and inhibitor susceptibility in *Chlamydia trachomatis*

**DOI:** 10.1099/mic.0.049668-0

**Published:** 2011-09

**Authors:** Xiaofeng Bao, Niseema D. Pachikara, Christopher B. Oey, Amit Balakrishnan, Lars F. Westblade, Ming Tan, Theodore Chase, Bryce E. Nickels, Huizhou Fan

**Affiliations:** 1 Department of Physiology and Biophysics, Robert Wood Johnson Medical School, University of Medicine and Dentistry of New Jersey, Piscataway, NJ 08854, USA; 2 Laboratory of Molecular Biophysics, The Rockefeller University, 1230 York Avenue, New York, NY 10065, USA; 3 Department of Microbiology and Molecular Genetics, and Department of Medicine, University of California, Irvine, CA 92697, USA; 4 Department of Biochemistry and Microbiology, School of Environmental and Biological Science, Rutgers, The State University of New Jersey, New Brunswick, NJ 08901, USA; 5 Department of Genetics and Waksman Institute, The State University of New Jersey, Piscataway, NJ 08854, USA

## Abstract

*Chlamydia trachomatis,* an obligate intracellular bacterium, is a highly prevalent human pathogen. Hydroxamic-acid-based matrix metalloprotease inhibitors can effectively inhibit the pathogen both *in vitro* and *in vivo*, and have exhibited therapeutic potential. Here, we provide genome sequencing data indicating that peptide deformylase (PDF) is the sole target of the inhibitors in this organism. We further report molecular mechanisms that control chlamydial PDF (cPDF) expression and inhibition efficiency. In particular, we identify the σ^66^-dependent promoter that controls cPDF gene expression and demonstrate that point mutations in this promoter lead to resistance by increasing cPDF transcription. Furthermore, we show that substitution of two amino acids near the active site of the enzyme alters enzyme kinetics and protein stability.

## Introduction

*Chlamydia trachomatis* is one of two species of chlamydiae that naturally infect humans, and is arguably the number one sexually transmitted bacterial pathogen worldwide (Schachter, 1999). Sexually transmitted chlamydial infection may lead to cervicitis, endometritis and salpingitis in women, and urethritis and lymphogranuloma venereum in both men and women. In addition, some serovars are responsible for preventable blindness in developing countries (Schachter, 1999).

Chlamydiae have a unique developmental cycle that involves two cellular forms. The metabolically quiescent, infectious elementary body (EB) is taken into a vacuole designated an inclusion through host cell endocytosis. Inside the inclusion, the EB develops into the proliferative but non-infectious reticulate body (RB). As RBs accumulate, they differentiate back into EBs in an asynchronous manner (Moulder, 1991). At the end of the developmental cycle, EBs and residual RBs are released when the infected cell bursts.

In an attempt to investigate the mechanisms underlying chlamydial development, we assessed the effects of small compounds that target a variety of signalling pathways in host mammalian cells. This screen identified hydroxamate-based matrix/ecto-metalloprotease inhibitors, exemplified by *N*-[(2*R*)-2-(hydroxamidocarbonylmethyl)-4-methylpentanoyl]-l-tryptophan methylamide, designated GM6001, and *N*-(*R*)-[2-(hydroxyaminocarbonyl)methyl]-4-methylpentanoyl-l-naphthylalanyl-l-alanine amide, known as TAPI, as highly effective inhibitors of *C. trachomatis* infection without detectable toxicity to host cells (Balakrishnan *et al.*, 2006, 2009). Time kinetics revealed that the compounds do not inhibit chlamydial entry into the host cell. Therefore, the inhibitors might directly target a chlamydial gene product that is essential for chlamydial infection; alternatively, the activity of a host metalloprotease or another hydroxamate-inhibited host metalloenzyme may indirectly support chlamydial growth. Interestingly, whereas a point mutation has been identified in a 5′-non-coding region of the peptide deformylase (PDF) gene in a chlamydial variant designated GR10 that was selected to grow in the presence of GM6001, no mutations were found in the variant's metalloprotease genes (Balakrishnan *et al.*, 2006).

PDF is a metallohydrolase essential for protein maturation in lower organisms. Newly synthesized proteins in eubacteria are led by *N-*formylmethionine. In most cases, bacterial neopeptides are converted to mature proteins through the sequential removals of the *N-*formyl group and methionine by PDF and methionine aminopeptidase, respectively. In a small proportion of proteins, the initiator methionine is retained after the removal of the formyl group (Capecchi, 1966). Similar to the zinc-dependent matrix metalloproteases, PDF contains a metal-binding HEXXH (X, any amino acid) motif. Based on studies of PDFs from a limited number of species, it is generally thought that the enzyme uses ferric iron *in vivo* for catalysis (Rajagopalan *et al.*, 1997). However, recombinant PDFs with other divalent metal ions, cobalt, nickel and zinc, in place of iron can be prepared, although metal substitution may affect enzyme activity (Rajagopalan *et al.*, 1997); furthermore, a PDF using zinc as the native catalytic ion has been identified in an iron-deficient bacterium (Nguyen *et al.*, 2007). In addition to the correlation of the chlamydial PDF (cPDF) gene mutation with the GM6001-resistant phenotype, and the absence of any metalloprotease gene mutation, there was also an increased cPDF protein level in GR10. Furthermore, GM6001 and TAPI directly inhibited cPDF enzyme activity (Balakrishnan *et al.*, 2006). These findings prompted us to hypothesize that these compounds interfere with *C. trachomatis* growth by exclusively targeting cPDF.

Deletion of the PDF gene in *Escherichia coli* results in a lethal phenotype (Mazel *et al.*, 1994), indicating that PDF is a potential antibacterial target. Consistent with this notion, topical application of GM6001 significantly reduced the loading of *Chlamydia muridarum*, a mouse pathogen that models human chlamydial diseases, in mice that were vaginally infected (Balakrishnan *et al.*, 2009). In addition, other peptide deformylase inhibitors have been shown to inhibit chlamydiae *in vitro* (Apfel *et al.*, 2000; Wise *et al.*, 2002). The therapeutic and prophylactic potential of GM6001 and related compounds implicated by these findings highlights the necessity to determine whether they solely target cPDF and how the activity of this essential enzyme is regulated in *Chlamydia*, since answers to these questions are important for the design of new inhibitors with potential therapeutic value. Due to the lack of a methodology for gene transfer, we tested the single-target hypothesis by sequencing the whole genome of GR10, and investigated the mechanisms that regulate cPDF activity and inhibition efficiency by isolating additional resistant mutants. Our work concludes unambiguously that GM6001 and TAPI exclusively target cPDF in this pathogen. We also show that mutations in the 5′-non-coding region of the cPDF gene cause resistance to the inhibitors by elevating cPDF gene transcription through distinct mechanisms. Finally, we demonstrate that amino acid changes near the active site of the cPDF protein can lead to resistance by altering enzymic properties and protein stability.

## Methods

### 

#### Reagents.

GM6001 was purchased from Calbiochem. *N*-Formyl-methionine-alanine-serine (fMAS) was purchased from Bachem Biosciences. Total RNA isolation (TRI) reagent was purchased from Sigma-Aldrich Chemicals. The TALON metal affinity resin was purchased from Clontech. The enhanced chemiluminescence (ECL) and ECL Plus kits were purchased from Amersham. 2,4,6-Trinitrobenzenesulfonic acid (TNBSA) was purchased from Pierce. Taqman reverse transcription kit and SYBR Green PCR Core kit were purchased from Applied Biosystems. *E. coli* RNA polymerase holoenzyme saturated with σ^70^ (eRNAP) and the core enzyme lacking any σ factor (eCore) were purchased from EPICENTRE. eRNAP lacking the C-terminal domain of the α subunit (ΔCTD) was purified using a novel method that enables isolation of *in-vivo*-assembled, recombinant eRNAP holoenzyme lacking amino acid residues 236–329 of the α subunit (Twist *et al.*, 2011). Monoclonal antibodies against chlamydial major outer-membrane protein (MOMP) and lipopolysaccharide (LPS) (Greene *et al.*, 2004; Xiao *et al.*, 2004) and a polyclonal mouse anti-cPDF (Balakrishnan *et al.*, 2006) have been described previously and were generous gifts from Dr Guangming Zhong (University of Texas Health Sciences Center at San Antonio).

#### Strains and culture conditions.

*C. trachomatis* serovar L2 (L2, strain 434/bu) was purchased from the American Type Culture Collection (ATCC) and expanded using HeLa cells as the host (Balakrishnan *et al.*, 2006). GR10 is an L2 variant that was selected for resistance to GM6001 as we reported previously (Balakrishnan *et al.*, 2006). Selection of Z1 was carried out with essentially the same procedures as for that of GR10 except that an ethyl methanesulfonate-mutagenized L2 stock was used as a starting material. Detection of chlamydial inclusions and determination of EB titres were done as detailed previously (Balakrishnan *et al.*, 2006). ArcticExpress *E. coli* was purchased from Stratagene. *E. coli* BW25113 substrains carrying wild-type or mutated cPDF alleles in place of the bacterial endogenous PDF gene (i.e. PDF-replacement *E. coli*) were constructed with phage λ Red recombinase technology (Datsenko & Wanner, 2000). Luria–Bertani (LB) medium (broth and 1.2 % agar plates) was used to culture *E. coli*. The MIC of GM6001 for *E. coli* was determined by inoculating exponential-phase bacteria on agar plates (8000 bacteria per plate) containing 1 : 2 serially diluted inhibitor and culturing the plates at 37 °C for 48 h.

#### RNA extraction and purification.

HeLa cells were infected with chlamydial stocks at three multiplicities of infection. At 24 h after infection, total cellular and chlamydial RNA was extracted with the TRI reagent following the manufacturer's instructions and treated twice with RNase-free DNase (Akers & Tan, 2006). To ensure that the RNA preparations were free of genomic DNA contamination, PCRs were performed to test whether MOMP and cPDF gene fragments could be amplified directly from the RNA. While reactions using 1 ng control genomic DNA showed amplification of expected DNA bands, those with 10 µg RNA samples prepared with double DNase digestions did not, confirming complete removal of genomic DNA from the RNA preparations.

#### cDNA preparation.

MOMP and cPDF cDNAs were synthesized by using gene-specific primers (MOMP, 5′-CAGCTGCGTTACAGAGAA-3′; cPDF, 5′-CATGCATTACAATGCTTGCTA-3′) and the Taqman Reverse Transcription kit, following the manufacturer's instructions.

#### Quantitative PCR (qPCR).

qPCR was performed on an Opticon real-time PCR machine (MJ Research) using the SYBR Green PCR Core kit. The 5′ primers for cPDF and the reference MOMP gene were 5′-GAGGTAGCTAGACCCGATAAG-3′ and 5′-GAGTGCTGGAGCTCGTGC-3′, respectively. The 3′ primers were the same as those for cDNA synthesis described above.

#### Western blotting.

Chlamydia-infected HeLa cells were lysed in SDS-PAGE gel loading buffer at 24 h after infection. PDF-replacement *E. coli* cultured in LB broth was harvested in the same buffer when the OD_600_ reached 0.8. The extracts were further sonicated. MOMP and cPDF were detected by ECL and ECL Plus, respectively. The signal intensities of the protein bands were quantified by densitometry (Balakrishnan *et al.*, 2006).

#### Preparation of crude genomic DNA for PCR and Sanger sequencing.

Chlamydiae were released from cells 40 h after infection by replacing the culture medium with water and incubating at room temperature for 10 min. Following centrifugation (500 ***g****,* 15 min) to remove cell debris, chlamydiae were lysed in buffer containing SDS and Triton X-100, and subjected to overnight digestion with proteinase K (Balakrishnan *et al.*, 2006). Nucleic acids were purified by phenol/chloroform extraction.

#### Sequencing of the cPDF gene.

Primers for amplifying and sequencing the cPDF gene as well as a portion of each of the neighbouring CTL0606 (corresponding to CT352 of *C. trachomatis* D) and CTL0608 (corresponding to CT354) have been reported (Balakrishnan *et al.*, 2006). The DNA fragment was PCR-amplified, gel-purified and subjected to automated sequencing based on fluorochrome-conjugated dideoxynucleotide termination. The amplification primers were used for sequencing.

#### Preparation of highly purified chlamydial genomic DNA for whole genome sequencing.

HeLa cells were infected with GR10. Chlamydiae were released from infected cells as described above. Host DNA and RNA were removed by incubating the lysates at 37 °C for 30 min after the addition of DNase and RNase. EBs in the lysates were purified by ultracentrifugation through 40 %/44 %/52 % Renocal gradients (Caldwell *et al.*, 1981). Nucleic acids were extracted as described above. Highly purified chlamydial DNA was obtained following RNase digestion, phenol/chloroform extraction and ethanol precipitation.

#### Full genome sequencing.

Genome sequencing, including library preparation, Solexa cluster generation and single-end-read sequencing, was performed by Creative Biolabs.

#### Production of His-tagged cPDF.

pET21-based vectors for expressing F134C, R137S and F134C/R137S cPDF carrying a carboxyl-terminal His_6_-tag were constructed similarly to that for wild-type cPDF as previously described (Balakrishnan *et al.*, 2006). Production and purification of the recombinant proteins were carried out following published procedures with modification (Balakrishnan *et al.*, 2006). Briefly, plasmid-transformed ArcticExpress *E. coli* was cultured on a shaker at 30 °C. When the OD_600_ of the culture reached ~0.8, the culture temperature was lowered to 13 °C. IPTG was added to the culture (final concentration: 1 mM) to induce recombinant gene expression. After overnight culture at 13 °C, the bacteria were collected by centrifugation and lysed by a French press. Cell debris was removed by centrifugation at 25 000 ***g***bold> for 30 min. cPDFs were purified with the TALON affinity metal resin and were stored at −80 °C after the addition of glycerol to a final concentration of 10 % (v/v).

#### cPDF activity assay.

An assay previously developed for the *E. coli* PDF (Groche *et al.*, 1998) was modified to measure the activity of the chlamydial enzyme. The assay mix, in a total of 50 µl reaction volume, contained 50 mM HEPES (pH 7.2), 10 mM NaCl, 125 ng cPDF, 0–50 mM fMAS and 50–500 nM GM6001. The deformylation reaction was allowed to proceed at 37 °C for 10 min and then terminated by heating at 95 °C for 2 min. The amount of MAS produced was reported by TNBSA, which reacts with the free amine group to form a chromogenic peptide conjugate. Briefly, 550 µl 0.0036 % TNBSA, freshly prepared in 0.1 M NaHCO_3_, was added to the reaction and incubated at 37 °C for 1 h. Following the addition of 200 µl 10 % SDS and 100 µl 1.0 M HCl, the peptide–TNBSA conjugate was quantified by measuring *A*_335_. All *A*_335_ values were within the range of free methionine standards. Catalytic constants were calculated from Lineweaver–Burk plots. Data presented were calculated from three experiments, except the *K*_i_ for the F134C/R137S cPDF, which was calculated from two experiments.

#### Preparation of chlamydial RNA polymerase (cRNAP).

cRNAP was prepared from L2-infected suspension culture of mouse L cells using heparin-conjugated agarose as previously described (Tan & Engel, 1996).

#### Preparation of recombinant chlamydial σ^66^.

The ORF of σ^66^ of L2 was cloned into the pET28 vector, designed for expression of amino-terminally His-tagged proteins using *Nde*I and *Xho*I cutting sites. Expression of the recombinant σ^66 ^in *E. coli* and purification using the TALON affinity metal resin were carried out as described for His-tagged cPDF.

#### *In vitro* transcription assay.

The abilities of DNA fragments containing candidate promoter sequences to direct RNA synthesis were determined using an *in vitro* transcription assay previously described (Tan & Engel, 1996). Briefly, the fragments were cloned into the transcription vector pMT1125 (Yu *et al.*, 2006) between the *Xba*I and *Eco*RV cutting sites. Immediately downstream of the *Eco*RV site in pMT1125 is a 154 bp G-less transcription cassette (Yu *et al.*, 2006). The assay in a total volume of 30 µl contained 300 ng supercoiled plasmid DNA, 1 mM potassium acetate, 8.1 mM magnesium acetate, 50 mM Tris/acetate (pH 8.0), 27 mM ammonium acetate, 1 mM dithiothreitol, 3.5 % (v/v) polyethylene glycol (average molecular mass, 8000), 330 µM ATP, 330 µM UTP, 1 µM CTP, 0.21 µM [α-^32^P]CTP [3000 Ci mmol^−1^ (111 TBq mmol^−1^, 100 µM 3′-*O*-methyl-GTP, 36 units of RNasin and RNA polymerase [i.e. cRNAP, eRNAP (*E. coli* RNAP holoenzyme) or eCore (*E. coli* RNAP core enzyme free of σ^70^) plus recombinant chlamydial** σ^66^]. The reaction was allowed to proceed at 37 °C for 30 min and terminated by the addition of 70 µl 2.86 M ammonium acetate containing 4 mg glycogen. After ethanol precipitation, ^32^P-labelled RNA was resolved by urea-PAGE and visualized on a Storm Phosphorimager (Molecular Dynamics), and the intensities of the 157 nt transcript bands were determined by the ImageQuant software. Data presented in bar graphs are means±sd of three experiments.

## Results

### The cPDF gene is the only gene mutated in the chlamydial variant GR10

The identification of a point mutation in the cPDF gene in strain GR10, selected for resistance to GM6001 and TAPI, suggests that these compounds target cPDF even though they are best known as metalloprotease inhibitors (Balakrishnan *et al.*, 2006). Nevertheless, the lack of a genetic system to conveniently manipulate *C. trachomatis* makes it difficult to rule out the possibility that they may have an additional target in this organism. To address this concern, the whole genome of GR10 was sequenced using the Solexa platform (Bennett, 2004). The genome resequencing project generated a total of 7 825 806 reads. Among these, 7 450 375 reads were matched to the reference L2 genome (Thomson *et al.*, 2008), whereas the remaining 375 431 (4.8 % of the total) reads represented contaminating DNA sequences from the host cells. The average coverage depth was 256×, leaving no unsequenced gap in the 1 038 842 bp genome (Thomson *et al.*, 2008). As expected, Solexa sequencing identified the known C→A mutation in the 5′-non-coding region of the cPDF gene, corresponding to nucleotide 723 246 in the reference genome (Thomson *et al.*, 2008). No additional mutations were found in the GR10 genome. Thus, cPDF is the authentic and sole target of GM6001 and TAPI in chlamydiae. Furthermore, we performed qPCR analysis using primers targeting the cPDF and the MOMP genes to determine if the mutated cPDF gene had undergone amplification in GR10. The signal ratios of cPDF : MOMP were the same between GR10 and wild-type L2 (data not shown). Since these two genes are ~350 kb away from each other, the probability for the two genes to have coamplified is very low. Thus, qPCR eliminated the possibility of PDF gene amplification as a mechanism for resistance to PDF inhibitors, which had been observed in *Haemophilus influenzae* (Dean *et al.*, 2007).

### C→A mutation in GR10 increases cPDF gene transcription

**Fig. 1.**
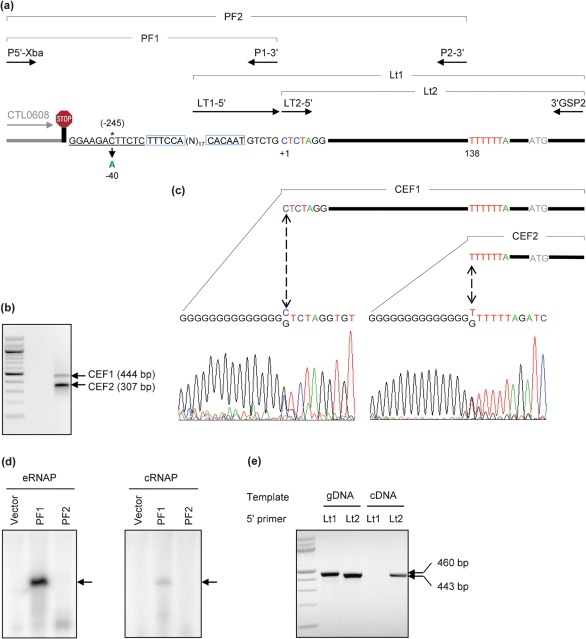
The GR 10 mutation was mapped to 40 nt upstream of the cPDF gene transcription start site. (a) Schematic drawing of the 5′-non-coding region of the cPDF gene, the 3′-end of the neighbouring CT0608 gene and locations of primers used for 5′-RACE and the confirmation of transcription start site. See Table S1 for positions of the genes and related fragments in the genome as well as the sizes of the fragments, and Table S2 for details of primers used. The stop sign signifies the Rho-independent transcription termination signal of CTL0608. Note that the fragment sizes are not to scale, and the gene fragment of CTL0608 is shown in halftone. The position of the C→A mutation in GR10 relative to the translation initiation site and the transcription start site as determined in panels (b–e) are numbered with and without parentheses, respectively. Putative −35 and −10 promoter elements are boxed and the UP element underlined. (b) Two cDNA 5′-end fragments, CEF1 and CEF2, corresponding to fragments in the cPDF gene drawn in (a), were detected by 5′-RACE using the primer 3′GSP2. In the left lane are 100 bp DNA markers; the lowest band is 100 bp. The indicated fragment sizes do not include the extra G : C pairs attached by the 5′-RACE reaction. (c) Sequencing trace forms showing 5′-ends of CEF1 and CEF2. Bidirectional arrows point to the 5′-end of the cDNAs. (d) *In vitro* RNA synthesis directed from the PF1 promoter fragment, which ends upstream of the 5′-end of CEF1, but not from PF2, which ends upstream of CEF2. Arrows point to the 157 nt transcript. See (a) for locations of PF1 and PF2 in the genomic DNA and Table S1 for details of primers (P5′-Xba, P1-3′ and P2-3′) used to clone PF1 and PF2 into pMT1125 (Yu *et al.*, 2006). (e) Amplification of cPDF cDNA using a 5′-end primer (Lt2) with sequence corresponding to the end of CEF1, but not a primer (Lt1) with sequence immediately upstream of the CEF1. The 3′ primer for all PCRs was 3′GSP2 (Table S2). Genomic DNA (gDNA) was used as control. Left lane: molecular markers as in (b).

The mutation in GR10 is located 245 nt upstream of the ATG initiation codon of the cPDF ORF and 7 nt downstream of the Rho-independent transcription termination signal of the neighbouring CTL0608 gene (Fig. 1a; see also Supplementary Table S1, available with the online version of this paper), suggesting that the mutation confers GM6001/TAPI resistance by increasing the cPDF gene expression. To test this hypothesis, we set out to identify the transcription initiation site in the gene. 5′-Rapid amplification of cDNA ends (RACE) using the 3′GSP2 primer (see Supplementary Table S2) yielded two cDNA end fragments, designated CEF1 and CEF2 (Fig. 1a,b; Supplementary Table S1). Sequencing analyses revealed that CEF1 started at 40 or 41 nt downstream of the mutation site of GR10, and CEF2 began at 137 or 138 nt downstream (Fig. 1c; Supplementary Table S1).

Between the mutation and the CEF1 initiation site there is a fragment with the sequence TTTCCA-(n)_17_-CACAAT (Fig. 1a) that loosely resembles the consensus σ^66^ promoter sequence, TTGACA-(n)_~17_-TATAAT (Tan, 2007). In contrast, no apparent promoter elements are readily recognized in the fragment upstream of the CEF2 initiation site. Since many chlamydial genes do not have consensus promoter elements (Tan, 2007), we used *in vitro* transcription assays to determine whether either or both upstream DNA fragments have promoter activity. While both the commercial eRNAP and cRNAP, purified from RBs, directed the synthesis of a reporting G-less RNA product from the candidate promoter fragment PF1, a genomic DNA fragment upstream of CEF1, neither RNAP synthesized RNA from the expanded promoter fragment PF2, which contained the additional 137 nt upstream of CEF2 (Fig. 1d and Supplementary Table S1). As expected, no transcriptional product was detected from the control promoter-less vector using either RNA polymerase. These results suggest that the smaller cPDF mRNA fragment that gave rise to CEF2 in 5′-RACE is a degradation intermediate of the full-length cPDF mRNA.

To determine if CEF1 might also correspond to a degradation intermediate of the cPDF mRNA, we attempted to amplify cPDF cDNA from the whole cDNA preparation using two alternative 5′-primers. One of these, Lt2-5′, targeted the 5′-end of CEF1, while the other, Lt1-5′, targeted a sequence immediately upstream of the 5′ end of CEF1 (Fig. 1a; Supplementary Table S1). Use of either Lt2-5′ or Lt1-5′ in conjunction with the 3′-primer 3′GSP2 amplified the expected 443 bp or 460 bp fragments when genomic DNA was used as template. In contrast, when cDNA was used as a template use of Lt2-5′ in conjunction with 3′GSP2 amplified the expected 443 bp product (Fig. 1e) whereas use of Lt1-5′ in conjunction with 3′GSP2 did not generate a product (Fig. 1e), suggesting that CEF1 is not a degradation intermediate. Taken together, the data presented in Fig. 1 indicate that the cPDF gene transcription is initiated at 40 or 41 nt downstream of the C→A mutation in GR10. This transcription start site is 5 nt earlier than the one reported in a transcriptome study using deep RNA sequencing (Amero *et al.*, 2009).

**Fig. 2.**
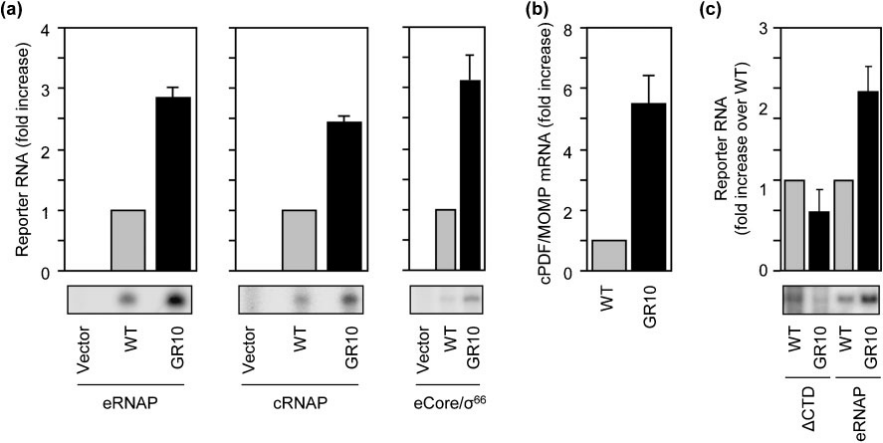
The non-coding nucleotide mutation in GR10 increases cPDF gene transcription by stabilizing promoter–αCTD interactions. (a) Promoter fragment containing the GR10 mutation showed increased transcription activity, as compared with the wild-type promoter fragment, in *in vitro* transcription assays using either *E. coli* RNAP holoenzyme (eRNAP), chlamydial RNAP (cRNAP) or the hybrid eCore/σ^66^ holoenzyme made of the *E. coli* RNAP core enzyme (eCore) and recombinant chlamydial σ^66^. Bands and quantitative data of the 154 nt ^32^P-labelled report transcript are shown. (b) Overexpression of the cPDF mRNA in GR10 as demonstrated by qPCR. (c) The GR10 mutation failed to stimulate transcription *in vitro* using eRNAP lacking the αCTD (ΔCTD). Data represent means±sd of three or more experiments.

Next, we determined the effect of the GR10 mutation on transcription. To do this, we compared the yields of transcripts generated *in vitro* in reactions performed using either the wild-type PF1 template or a template carrying the GR10 mutation. We found that introduction of the GR10 mutation into the PF1 template resulted in a 2.8- and 2.4-fold increase in yields of full-length transcripts, in reactions done with eRNAP (Fig. 2a, left) and cRNAP (Fig. 2a, middle), respectively. A 3.1-fold increase in transcription was also detected using the σ^70^-free eCore in the presence of recombinant chlamydial σ^66^ (Fig. 2a, right). Consistent with these *in vitro* transcription data, reverse transcriptase qPCR analysis detected a 5.5-fold increase in the abundance of cPDF mRNA, relative to the mRNA level of MOMP, in GR10, as compared with wild-type L2, at 24 h after infection (Fig. 2b), which is consistent with our previous analysis demonstrating a comparable degree of elevation in the level of cPDF protein (Balakrishnan *et al.*, 2006).

In addition to the recognition of the −35 and −10 promoter elements by σ^66^, promoter binding also involves interactions between the C-terminal domain of the RNAP α subunit (αCTD) and sequences immediately upstream of the promoter −35 element (Haugen *et al.*, 2008). Furthermore, at certain promoters (most notably the ribosomal promoters) the αCTD makes sequence-specific contact with an A+T-rich sequence element, called the UP element. Given that the GR10 mutation increases the A+T content of the DNA upstream of the putative −35 hexamer (Fig. 1a), we hypothesized that the GR10 mutation increases transcription from the cPDF promoter by stabilizing interactions between the αCTD and upstream DNA. To test this hypothesis we performed *in vitro* transcription reactions using RNAP lacking the αCTD. In this case, yields of full-length transcripts were essentially identical in reactions performed using the wild-type template or reactions performed using the template carrying the GR10 mutation (Fig. 2c). These findings are consistent with the hypothesis that the GR10 mutation increases transcription from the cPDF promoter by stabilizing interactions between the αCTD and upstream DNA.

### Identification of four point mutations in the cPDF gene of resistant variant Z1

**Fig. 3.**
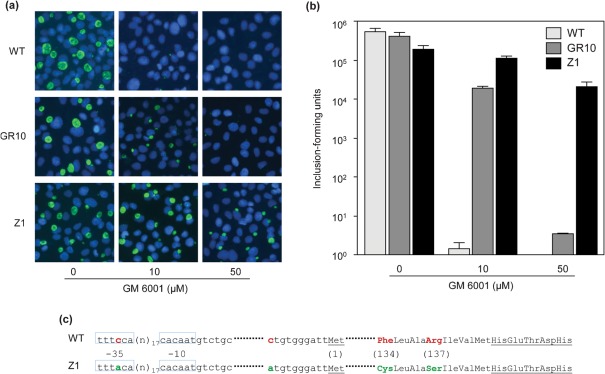
Resistance to GM6001 and cPDF gene mutations in the Z1 variant. (a, b) HeLa cell monolayers were infected with an EB stock of wild-type *C. trachomatis,* GR10 or Z1 and were cultured with medium containing the indicated concentrations of GM6001. (a) Immunofluorescence microscopy was performed 24 h after infection using the anti-LPS antibody and a FITC-conjugated secondary antibody to visualize inclusions (green). DAPI was used to counterstain DNA (blue). Experiments were done twice and yielded similar results. (b) Progeny EB production (means±sd of two experiments) was determined 40 h after infection by quantifying inclusions formed in secondary cultures. (c) Schematic presentation of four mutations in the cPDF gene of Z1. Mutated nucleotides and amino acids in Z1 as well as their wild-type counterparts are shown in colour. Putative −35 and −10 promoter elements are shown in boxes. Amino acids are numbered in parentheses. The leading methionine and metal-binding HEXXH motif are underlined.

GM6001 not only inhibits chlamydial growth in cell culture, but also can reduce vaginal chlamydial loading in a mouse model (Balakrishnan *et al.*, 2009). This suggests that PDF inhibitors may be useful for prevention and treatment of chlamydial infection. Therefore, we sought to identify additional mechanisms that control cPDF activity, as such investigation may help the design of cPDF-targeting drugs. We approached this by selecting and characterizing new GM6001-resistant mutants. To increase the efficiency of mutant selection, we performed random mutagenesis using the DNA-damaging reagent ethyl methanesulfonate. From a mutagenized L2 stock, we obtained a GM6001-resistant mutant designated Z1. Compared with GR10, Z1 exhibited an even stronger tolerance to GM6001, as indicated by immunofluorescence microscopy detecting chlamydial inclusions formed in the presence of GM6001 (Fig. 3a) and by the enumeration of progeny EBs produced by GM6001-treated cultures (Fig. 3b).

A 1142 bp genomic DNA fragment that covers the entire cPDF gene (i.e. CTL0607, coding and non-coding regions) and an approximately 100 bp region of each of the two neighbouring genes (i.e. CTL0606 and CTL0608) of the Z1 variant was sequenced, which revealed four point mutations in the cPDF gene (Fig. 3c). Two of the mutations were found in the 5′-non-coding region, one at nucleotide −31 relative to the transcription start site and the other at −10 relative to the translation initiation codon. The remaining two point mutations were found in the coding region, leading to two amino acid substitutions (F134C and R137S) in the protein.

### A putative −35 promoter element mutation increases cPDF transcription

The locations of the mutations suggest that a number of mechanisms contribute to the resistance in Z1. First, the most upstream mutation may increase transcription by enhancing the σ^66^ binding since the mutation lies in the putative −35 promoter element and the new TTTACA hexamer in Z1 is predicted to be a more favourable hexamer for σ^66^ as compared with the wild-type TTTCCA sequence, based on findings from an *in vitro* mutational analysis of the *dnaK* promoter (Schaumburg & Tan, 2003). For the convenience of description, this mutation is designated Z1P−35. Second, since the mutation near the translation start site is in the ribosomal binding site (RBS) in mRNA, it might increase protein translation efficiency. Indeed, a similar mutation has previously been reported to be responsible for the overexpression of ribonucleotide reductase small subunit and thus for increased resistance to hydroxyurea, an inhibitor of the reductase, in *C. trachomatis* (Roshick *et al.*, 2000). Third, since F134 and R137 are near the essential metal-binding HEXXH motif of cPDF (Fig. 3c), the F134C and R137S substitutions probably alter the enzyme activity.

**Fig. 4.**
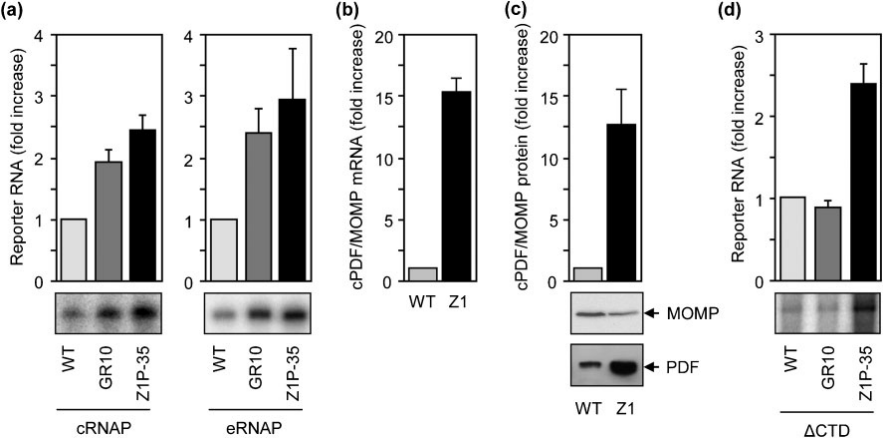
The Z1P−35 mutation increases transcription from the cPDF promoter. (a) Transcription from PF1 (Fig. 1) harbouring the Z1P−35 mutation or GR10 mutation was increased, as compared with that from wild-type PF1, using cRNAP or eRNAP. (b, c) Increased cPDF mRNA and protein, respectively, were detected in Z1 as compared with wild-type *C. trachomatis.* (d) The transcription-stimulatory activity of the Z1P−35 mutation but not the GR10 mutation was detectable using ΔCTD. Experiments in (a–d) were performed as in Fig. 1. Data represent means±sd of three or more experiments.

We performed *in vitro* transcription to test the hypothesis that the Z1P−35 mutation increases the cPDF gene transcription. Both cRNAP and eRNAP synthesized higher levels of full-length transcripts from PF1 harbouring the mutation, as compared with the wild-type PF1 (Fig. 4a). Consistent with the *in vitro* transcription data, reverse transcriptase qPCR detected a 15-fold higher amount of the cPDF mRNA in the Z1 mutant (Fig. 4b); furthermore, Western blotting revealed a similar increase in the level of cPDF protein (Fig. 4c).

Since the Z1P−35 mutation is located in the putative −35 hexamer, which binds σ^66^, we hypothesized that the ability of the mutation to increase transcription is not affected by the deletion of the αCTD. Indeed, the Z1P−35 mutation also increased levels of full-length transcripts in reactions performed with ΔCTD (Fig. 4d). As the αCTD-less polymerase failed to produce an increased amount of full-length transcripts from the PF1 template carrying the GR10 mutation (Figs 2c and 4d), the GR10 and Z1P−35 mutations upregulate cPDF gene transcription through distinct mechanisms.

### F134C and R137S substitutions and non-coding mutations contribute to resistance

**Fig. 5.**
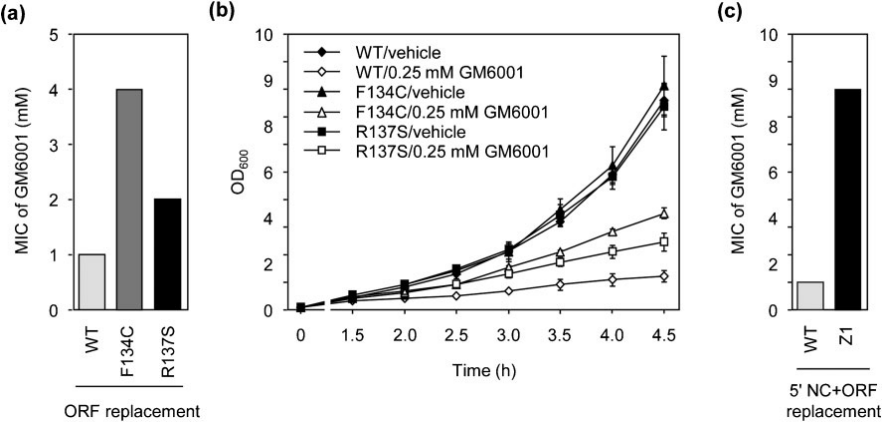
Effects of F134C, R137S and non-coding mutations found in Z1 on GM6001 resistance in *E. coli.* (a) PDF replacement *E. coli* clones carrying the ORF for the F134C or the R137S cPDF displayed increased MICs as compared with wild-type ORF-replacement clones. MIC was determined on agar culture plates with 1 : 2 serially diluted GM6001. (b) F134C and R137S ORF-replacement *E. coli* exhibited high levels of resistance to 0.25 mM GM6001 in liquid culture medium as compared with wild-type ORF-replacement bacteria. Growth was assessed by OD_600_. Data are means±sd of three experiments. (c) Concurrent replacement of the Z1 mutant's ORF and 5′ non-coding sequence (5′NC) in *E. coli* resulted in an increased MIC for the inhibitor. In (a) and (c), for each replacement, three clones were analysed and had the same MIC.

We next determined the roles of F134C and/or R137S in resistance. The lack of a method to genetically manipulate *C. trachomatis* made it impossible to directly assess the role of each of the mutated residue in the resistance. To circumvent this technical difficulty, we set out to construct *E. coli* bearing the ORF of wild-type, F134C, R137S or the F134C/R137S cPDF in place of the endogenous PDF ORF using the phage λ Red recombinase methodology (Datsenko & Wanner, 2000). We obtained multiple *E. coli* clones with F134C, R137S or wild-type ORF replacement after a single round of engineering. PCR was performed using primers targeting the flanking sequences of the *E. coli* PDF ORF to confirm the replacement (data not shown). However, we were unable to construct replacement *E. coli* bearing the F134C/R137S ORF despite multiple attempts. The abilities of the F134C and R137S replacement bacteria (three clones each) to grow in the presence of GM6001 were determined. Consistent with a previous report from our lab, *E. coli* is intrinsically much more resistant to GM6001 than chlamydiae (Balakrishnan *et al.*, 2006): the MIC of GM6001 is at millimolar level in the enterobacterium and in the micromolar range in chlamydiae. Nevertheless, the effects of different cPDFs on the tolerance to GM6001 in *E. coli* were evident on agar plates. Thus, whereas bacteria expressing the F134C cPDF resisted a fourfold higher GM6001 concentration as compared with the bacteria expressing wild-type cPDF, those expressing the R137S cPDF also exhibited a twofold higher tolerance (Fig. 5a). Furthermore, growth curves established in liquid medium containing 0.25 mM GM6001, which is below the MICs, also showed higher levels of resistance to GM6001 in the F134C and R137S replacement *E. coli* (Fig. 5b).

Our failure to replace the ORF of the endogenous PDF of *E. coli* with that of the F134C/R137S cPDF suggests that the mutant chlamydial enzyme is unable to replace the endogenous *E. coli* PDF. Nevertheless, when the 5′ non-coding region containing the Z1P−35 and the RBS mutation from Z1 was included in the targeting allele, we readily obtained replacement bacteria, as confirmed by genomic PCR analysis (data not shown). The MICs of GM6001 for three replacement *E. coli* clones containing the quadruple mutations were 8 mM, whereas those for the three replacement clones carrying the 5′ non-coding region and the ORF of wild-type cPDF were 1 mM. These data further illustrate the roles of the 5′ non-coding mutations in cPDF overexpression and GM6001 resistance in Z1 (Fig. 5c).

### Changes in kinetic properties in F134C, R137S and F134C/R137S cPDFs

To assess the effects of F134C and R137S substitutions on enzyme properties, we expressed cPDF with either or both substitutions as well as wild-type cPDF protein in *E. coli* and purified them to near homogeneity (data not shown). With fMAS as a substrate, the F134C and R137S single mutants both had a nearly twofold higher *K*_m_ as compared with the wild-type enzyme, whereas the double mutants had a nearly 10-fold larger *K*_m_ (Table 1). All the mutant enzymes had increased *k*_cat_ values over the wild-type enzyme (Table 1). The two single mutants exhibited increased *k*_cat_/*K*_m_ values whereas the double mutant has a significant 27 % decrease in the *k*_cat_/*K*_m_ value, as compared with that of wild-type (Table 1). The *K*_i_ of GM6001 for the F134C/R137S cPDF variant was 6.3-fold higher than that for wild-type (Table 1). In comparison, the two single cPDF mutants had *K*_i_ values similar to the wild-type enzyme (Table 1).

**Table 1.** Changes in catalytic constants in cPDF loop mutants Data are means±sd.

Catalytic constant	Wild-type	F134C	R137S	F134C/R137S
*K*_m_ for fMAS (mM)	5.0±0.6	9.2±0.5**	9.3±1.1**	48.0±1.9**
% of control	100	184	186	965
*k*_cat_ (s^−1^)	6.4±0.5	19.6±2.0**	22.1±2.1**	45.3±4.2**
% of control	100	306	344	705
*k*_cat_*/K*_m_ (M^−1^ s^−1^)	1296±79	2143±215**	2387±85**	948±124*
% of control	100	165	184	73
*K*_i_ for GM6001 (nM)	160±35	143±0.1	148±41	1000±11**
% of control	100	89.3	92.6	625.3

* *P*<0.05, **P<0.01 (two-tailed Student's *t* test for comparison with the wild-type enzyme).

### F134C and R137S mutations affect cPDF protein stability

**Fig. 6.**
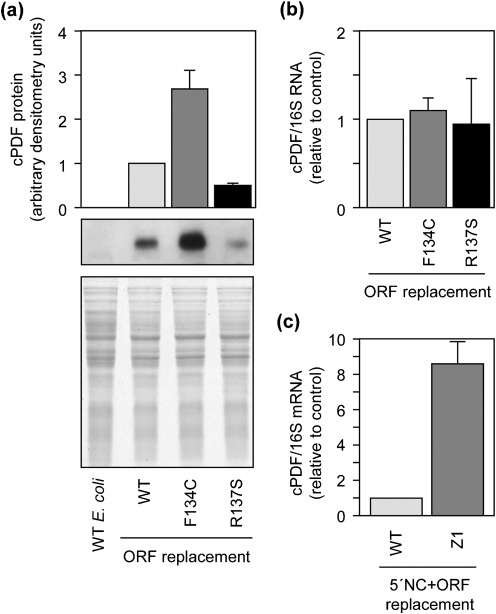
Effects of F134C and R137 mutations on cPDF protein expression in *E. coli.* (a) Western blot showing increased cPDF expression in F134C ORF replacement bacteria and decreased expression in R137S replacement. The bottom panel shows Coomassie blue staining of total resolved proteins. (b) qPCR detected similar amounts of cPDF mRNA among different replacement *E. coli* strains. (c) qPCR detected a higher amount of cPDF mRNA in the replacement *E. coli* carrying all mutations found in the cPDF gene of Z1, as compared with the replacement bacteria harbouring the 5′-non-coding region and ORF of wild-type *C. trachomatis.* Data in (a–c) are representative of three or more experiments.

As shown in Fig. 5, the two singly mutated PDF proteins exhibited very similar (if not identical) enzymic kinetics. However, in gene targeting experiment, the F134C cPDF conferred a twofold higher resistance to GM6001 than the R137S form in *E. coli*. To resolve this discrepancy, we determined the expression levels of the two mutants in bacteria by Western blot analyses. Interestingly, the expression level of F134C cPDF is higher than that of the wild-type protein, while that of the R137S is lower (Fig. 6a). A duplicate gel, stained with Coomassie blue, showed equal amounts of protein loadings among the replacement mutants (Fig. 6a, bottom). Western blotting analysis showed that the cPDF antibody recognized the two forms of purified His-tagged cPDF proteins equally efficiently (data not shown).

Analyses were performed to probe possible mechanisms leading to the variation in protein expression of the F134C and R137S cPDFs. First, the promoter region of the ORF replacement *E. coli* was sequenced, and no mutation was detected. Second, the amounts of cPDF mRNA relative to the 10S rRNA in the three ORF replacement bacteria were determined and found to be comparable (Fig. 6b). As expected, there was a large increase of cPDF mRNA in replacement *E. coli* carrying both the ORF and 5′ non-coding region of the cPDF of Z1, compared with the bacteria knocked-in with the corresponding wild-type sequences (Fig. 6c). Taken together, these results suggest that F134C and R137S substitutions affect cPDF protein stability.

## Discussion

Although *C. trachomatis* is susceptible to a number of antibiotics, the majority of infected individuals do not seek medical treatment. This is true even in places with good health care because the infection often generates very mild or no symptoms (Schachter, 1999). Without proper antibiotic treatment, more than one-third of infected cases develop serious complications, including infertility, ectopic pregnancy, abortion and chronic pelvic inflammatory pain syndrome (Schachter, 1999). Infected people are also at increased risk of HIV acquisition, due to ulcerative damage caused by *C. trachomatis* replication (Stamm *et al.*, 1988). Therefore, identification of potential new therapeutic and prophylactic targets in this organism is of high importance.

Our previous study on GR10 suggested that inhibition of cPDF rather than metalloproteases of either host or parasitic origin accounts for the antichlamydial effect of GM6001 (Balakrishnan *et al.*, 2006). Nevertheless, that study did not rule out the possibility that the inhibitor has another target in *Chlamydia.* In this work, full genome sequencing revealed the cPDF gene as the only gene carrying a mutation in GR10. The discovery of cPDF as the exclusive target of GM6001 in *Chlamydia* is of significance for the development of GM6001 derivatives for the treatment and prevention of chlamydial disease, and further raises the importance of understanding the regulation of cPDF activity.

The chlamydial genome encodes three σ factors: the primary σ factor σ^66^ and the alternative σ factors σ^28^ and σ^54^ (Stephens *et al.*, 1998). Our findings suggest that the cPDF gene is recognized by σ^66^. Thus, our cRNAP, which should contain only σ^66^ but not the two alternative σ factors according to previous studies (Tan, 2007), can transcribe from the cPDF promoter. In addition, the transcription from the putative promoter region is also detectable using a commercial *E. coli* RNAP holoenzyme containing σ^70^, a σ^66^ homologue with the capacity to functionally substitute for the chlamydial primary σ factor in a number of cases examined (Hefty & Stephens, 2007; Koehler *et al.*, 1990; Shen *et al.*, 2000; Tan *et al.*, 1998). Finally, the transcription was also evident with a hybrid RNAP holoenzyme in which recombinant σ^66^ was used in place of σ^70^.

Several lines of evidence indicate that the mutation in GR10 causes resistance to PDF inhibitors by stabilizing the interaction between the αCTD and DNA immediately upstream of the −35 element of the cPDF promoter. First, according to published work, the binding of the proximal UP element by αCTD is centred at around nucleotide −41 (Chen *et al.*, 2003; Ross *et al.*, 1998), which coincides with the mutation site in GR10. Furthermore, the C→A mutation in GR10 is predicted to result in more efficient αCTD binding because αCTD favours A+T-rich sequences (Chen *et al.*, 2003; Ross *et al.*, 1998). Finally, the transcription-enhancing activity of the GR10 mutation became completely undetectable when the intact eRNAP was replaced with ΔCTD.

Transcription analyses showed that, similar to the GR10 mutation, the Z1P−35 mutation identified in the Z1 resistant variant has a stimulatory effect on cPDF gene transcription. However, the GR10 and Z1P−35 mutations upregulate transcription by enhancing the interaction of different components of the transcription machinery. Whereas the Z1P−35 mutation is predicted to result in a more efficient −35 hexamer for σ^66^, the GR10 mutation increases the interaction between the αCTD and the DNA upstream of the −35 element. Previously, a number of chlamydial promoters and their interactions with the RNAP have been studied *in vitro* or with heterologous systems (reviewed by Tan, 2007). However, to the best of our knowledge, this study is the first one demonstrating that single nucleotide changes in promoter elements can affect drug susceptibility in *Chlamydia*.

Two other sites that affect GM6001 targeting efficiency are found in the cPDF protein: F134 and R137. The abilities of the F134C and R137S mutations to mediate resistance were confirmed using a heterologous system. Accordingly, replacement *E. coli* strains expressing either mutated cPDF exhibit a higher level of GM6001 tolerance as compared with bacteria expressing wild-type cPDF. Interestingly, each of the mutations alone has no negative effect on GM6001 binding; they contribute to resistance by increasing the catalytic efficiency. The improved catalytic efficiency in the R137S enzyme may be a key mechanism for resistance in its replacement *E. coli* strain since the protein is expressed less efficiently than wild-type enzyme. In comparison, an increased stability of the F134C protein, leading to a rise in the enzyme amount, is another contributory factor. In both F134C and R137S mutants, the elevations in *K*_m_ along with the increases in *k*_cat_ in the absence of concurrent increases in *K*_i_ suggest that product release and/or a conformational shift just prior to product release may be largely rate-limiting in wild-type cPDF and is accelerated in the mutant enzymes.

Compared with the F134C and R137S variants, the F134C/R137S cPDF displayed an over sixfold increase in *K*_i_ over wild-type and a nearly 10-fold elevation in *K*_m_. The reduced affinity to GM6001 would confer resistance to the inhibitor. Since there is also a 27 % decrease in *k*_cat_/*K*_m_, the large increase in *K*_m_ suggests an extensive loss in substrate-binding, which most probably has made the mutant cPDF an ineffective enzyme *in vivo.* The fact that the F134C/R137S ORF was not able to support *E. coli* growth until the 5′ non-coding region carrying the Z1P−35 and RBS mutations were knocked-in concurrently to increase its expression level supports this supposition. Finally, a significantly larger increase in *K*_m_ than that in *K*_i_ in the F134C/R137S cPDF further supports the hypothesis that product release and/or a conformational change before product release is rate-limiting in cPDF, as noted above.

Recently, homology modelling has localized F134 and R137 in an α-helix that contributes to the formation of the enzyme's active site (Oey *et al.*, 2011). Studies of PDFs from other organisms using X-ray crystallography and NMR spectroscopy have shown that binding of a ligand (i.e. substrate, product or inhibitor) to the enzyme induces conformational changes, particularly near the active site (Amero *et al.*, 2009; Guilloteau *et al.*, 2002). Furthermore, single amino acid substitutions proximal to the active site can result in changes in structure and ligand binding, leading to resistance (see structures 3M6O, 3M6P, 3M6Q and 3M6R at http://www.ncbi.nlm.nih.gov/structure). Similarly, our enzyme kinetic data suggest that F134 and R137 also regulate enzyme–ligand interactions and conformation during catalysis. Thus, the F134C and R137S mutations each alone reduce the affinity to the product, and in combination they also decrease substrate- and inhibitor-binding.

In summary, the findings of a mutation in the cPDF gene as the sole change in the GR10 genome as well as the correlation of cPDF gene mutations with resistance phenotype, cPDF gene expression and changes in kinetic properties in the Z1 variant represent definitive evidence for the targeting of cPDF by GM6001 in *C. trachomatis*. Analyses of protein and mRNA expression in the chlamydial variants, *in vitro* characterization of their promoters, kinetic characterization of recombinant enzymes and studies using the heterologous *E. coli* system indicate that the susceptibility of *C. trachomatis* to PDF inhibitors is controlled by cPDF gene transcription, protein expression and stability as well as catalytic properties of the cPDF enzyme. Since the Z1 mutant was obtained by random mutagenesis, it would not be surprising if mutations in other loci of the genome contribute to the resistant phenotype. Sequencing of the Z1 genome and characterization of additional mutated genes may further advance the understanding of mechanisms underlying the regulation of cPDF activity and susceptibility to PDF inhibitors, although separating genes with true regulatory functions from other mutated genes might prove challenging.

## Supplementary Material

Supplementary tables
