# Supplementary material for: Non-coding nucleotides and amino acids near the active site regulate peptide deformylase expression and inhibitor susceptibility in Chlamydia trachomatis
Source: Microbiology (Reading). 2011 Sep;157(Pt 9):2569–81. doi: 10.1099/mic.0.049668-0 (PMC3352175; doi:10.1099/mic.0.049668-0)
Supplement: Supplementary tables [file supp_157_9_2569__index.html]

Non-coding nucleotides and amino acids near the active site regulate peptide deformylase expression and inhibitor susceptibility in Chlamydia trachomatis — Supplementary tables 

# Non-coding nucleotides and amino acids near the active site regulate peptide deformylase expression and inhibitor susceptibility in *Chlamydia trachomatis*

### Non-coding nucleotides and amino acids near the active site regulate peptide deformylase expression and inhibitor susceptibility in *Chlamydia trachomatis*, by X. Bao, N. D Pachikara, C. B. Oey, A. Balakrishnan, L. F. Westblade, M. Tan, T. Chase Jr, B. E. Nickels and H. Fan

*Microbiology* vol. **157**, part 9, pp. 2569 - 2581

Supplementary tables [PDF] (87 kb):   
  
**Table S1.** Details of the cPDF gene and CTL0608 components and related materials   
  
**Table S2.** Sequences of primers used for identification and confirmation of the PDF gene transcription initiation site
