# Supplementary material for: Non-coding nucleotides and amino acids near the active site regulate peptide deformylase expression and inhibitor susceptibility in Chlamydia trachomatis
Source: Microbiology (Reading). 2011 Sep;157(Pt 9):2569–81. doi: 10.1099/mic.0.049668-0 (PMC3352175; doi:10.1099/mic.0.049668-0)
Supplement: Supplementary tables [file supp_157.9.2569_SupplementaryTables.pdf]

SUPPLEMENTARY TABLES

Noncoding nucleotides and amino acids near the active site regulate peptide deformylase expression and inhibitor susceptibility in *Chlamydia trachomatis*

Bao, X., Pachikara, N., Oey, C. B., Balakrishnan, A., Westblade, L. F., Tan, M., Chase, T., Nickels, B. E. and Fan, H.

Microbiology (2011), **157**, 2569–2581

Table S1. Details of THE cPDF gene and CTL0608 components and related materials

Start and end sites are numbered according to NCBI reference sequence NC_010280.1.

NCR, noncoding region.

Subject	Start	End	Length (bp)	Additional note
CTL0608 3′NCR	723314	723292	23	Based on prediction
PDF 5′NCR	723291	723246	46	
PDF ORF	723040	722495	546	
CEF1	723245	722802	444	Excluding bases added by the 5´RACE reaction
CEF2	723108	722802	307	Excluding bases added by the 5´RACE reaction
PF1	723389	723108	282	
PF2	723389	723244	146	
Lt1	323263	722802	460	
Lt2	323242	722802	443	

Table S2. Sequences of primers used for identification and confirmation of the PDF gene transcription initiation site

Start and end sites are numbered according to NCBI reference sequence NC_010280.1

Primer	Start	End	Sequence	Additional note
3′GSP2	722802	722822	5′-aataccctaggaaaatcacag-3′	
P5′-Xba	723389	723372	5′-*ttt*TCTAGAtcagagaagaccagaccc-3′	*Xba*I site in upper case; 5′-*ttt* was added to facilitate digestion during vector construction
P1-3′	723247	723264	5′-gagcagacattgtgatgg-3′	
P2-3′	723108	723324	5′-ttccctagcgtcttctg-3′	
Lt2-5′	323244	323225	5′-tctaggtgtcttggatttca-3′	
Lt1-5′	323263	323247	5′-tccatcacaatgtctgc-3′	
